# Extreme heat risk and the potential implications for the scheduling of football matches at the 2026 FIFA World Cup

**DOI:** 10.1007/s00484-025-02852-4

**Published:** 2025-01-25

**Authors:** Donal Mullan, Iestyn Barr, Neil Brannigan, Nuala Flood, Oliver R. Gibson, Catherine Hambly, Alan T. Kennedy-Asser, Aimée C. Kielt, Tom Matthews, Madeleine Orr

**Affiliations:** 1 School of Natural and Built Environment, Queen’s University Belfast, Belfast, Northern Ireland UK; 2 Department of Natural Sciences, Manchester Metropolitan University, Manchester, England, UK; 3 School of Geography and Environmental Sciences, Ulster University, Coleraine, Northern Ireland UK; 4 Department of Life Sciences, Brunel University London, London, England, UK; 5 School of Biological Sciences, University of Aberdeen, Aberdeen, Scotland, UK; 6 School of Geographical Sciences, University of Bristol, Bristol, England, UK; 7 Department of Geography, King’s College London, London, England, UK; 8 Faculty of Kinesiology and Physical Education, University of Toronto, Toronto, ON Canada

**Keywords:** Wet bulb globe temperature, Humidity, Exertional heat illness, Climate change, Sport, North America

## Abstract

Climate change is making extreme heat events more frequent and intense. This negatively impacts many aspects of society, including organised sport. As the world’s most watched sporting event, the FIFA World Cup commands particular attention around the threat of extreme heat. The 2022 tournament in Qatar was moved from summer to winter in response to this threat, and now attention turns to the 2026 tournament in North America with extreme heat risk across many of the 16 host locations. We examine this risk by modelling wet bulb globe temperature (WBGT) – a widely used measure of heat stress – for the host locations using hourly meteorological data for the period 2003–2022. Our results show that 14 out of 16 host locations exceed WBGTs of 28 °C, with four (nine) exceeding this threshold more than half the time during afternoons across the mean (hottest year) of the 20 year record. This threshold is important as the level beyond which some football governing bodies recommend match delay or postponement. A climatically sound argument is therefore presented to reschedule kick-off times outside the hottest afternoon hours for the host locations with highest heat risk at which no indoor air conditioned environment exists – primarily Miami and Monterrey, but also Philadelphia, Kansas City, Boston and New York. This study highlights the need to carefully assess heat risk ahead of major sporting events to help inform any potential interventions needed in the scheduling of matches and competitions in a warming climate.

**Supplementary Information:**

The online version contains supplementary material available at 10.1007/s00484-025-02852-4.

## Introduction

The frequency and intensity of hot extremes (including heatwaves) have increased on a global scale since 1950, and are projected to at least double at 2 °C warming and quadruple at 3 °C warming relative to present-day conditions (Seneviratne et al. 2021). These escalating heat extremes negatively impact many aspects of society, including organised sport. Perhaps most obviously in this context, extreme heat threatens athlete health owing to the elevated probability of exertional heat illness (EHI) during play (Garcia et al. 2022; Orr et al. 2022). These risks depend on environmental factors, e.g. hot temperatures, and personal factors, e.g. age, fitness, and existing health status (Roberts et al. 2021). EHI impacts a wide range of sports, with epidemiological studies reporting highest incidences in American football, running, cycling, and adventure races (Gamage et al. 2020). EHI takes many forms, including the life-threatening condition exertional heat stroke (EHS). EHS is characterised by central nervous system disturbances and hyperthermia, with body temperatures > 40 °C, leading to organ and tissue damage (Patel et al. 2023). Athlete deaths resulting from EHS have averaged three per year since 1995, mainly in American football high school players (Bruggers 2018). No documented cases of EHS or death have been reported among elite players in international football (soccer) (Mountjoy et al. 2012) – the focus of this study – but extreme heat has caused fatigue and injury, on-field collapses and hospitalisation in many professional players and match officials, including Houston Dash player Rachel Daly during a National Women’s Soccer League match against Seattle Reign in 2017 (ESPN 2017); Uganda captain Denis Onyango at the 2019 Africa Cup of Nations (AFCON) match against hosts Egypt; Nigeria player Samuel Kalu in training ahead of the 2019 AFCON (Edwards and Wachira 2019); Zambian referee Janny Sikazwe during a 2021 AFCON match between Tunisia and Mali in Cameroon (Independent 2022); and most recently Guatemalan match official Humberto Panjoj during a match between Canada and Peru at the 2024 Copa América in Kansas City, USA (Kloke and Burrows 2024). The above examples are not an exhaustive list, and it is important to note that EHIs are notoriously underreported as symptoms closely mirror other more minor health issues (e.g. Gibson et al. 2020). Beyond health, extreme heat impacts the physiological and technical performance of football players and match outcomes in mixed ways, including a reduction in total running distance and high intensity outputs in conditions of high vs. low environmental stress (e.g. Mohr et al. 2012; Nassis et al. 2015; Konefał et al. 2021); a 4% increase in peak sprint speed in hot ambient conditions (Mohr et al. 2012); a reduction in the sprint frequency of players in high vs. low and moderate heat stress (Nassis et al. 2015); an 8% and 9% increase in successful passes and crosses respectively in hot ambient conditions (Mohr et al. 2012); and a strong positive correlation between anomalous high temperatures and the number of penalty shootouts during the first knockout stage at FIFA Men’s World Cup events (*r* = 0.82) (Tobias et al. 2019).

Given the impacts of extreme heat on football in a warming climate, adaptive measures help ensure the safety of players and match officials, while also minimising any negative impact on player performance. Adaptation options include in-play cooling breaks (Chalmers et al. 2019), which are mandatory under FIFA heat policy when wet bulb globe temperature (WBGT) – a widely used measure of heat stress incorporating temperature, humidity, solar radiation and wind speed (Budd 2008) – reach or surpass 32 °C (Kramer and Dvořák, 2015). Adaptation options may also be technological, including the design of stadia to provide optimum shading, passive cooling and high albedo, while also integrating targeted air-conditioning technologies to cool the fans in the stands and the players on the field, e.g. the design of the 2022 World Cup stadia in Qatar (Lusweti and Odawa 2023). Adaptations may also be limited to the reorganisation of tournaments/matches to more climatically suitable locations or times of the day/year, e.g. the 2022 FIFA World Cup in Qatar was moved from June/July to November/December due to concerns around extreme heat (Borden 2015). Attention now turns to the risk of extreme heat at the upcoming 2026 FIFA World Cup hosted by USA, Canada and Mexico. Extreme heat has already impacted the 2024 Copa América tournament played during the same months and at many of the same venues (The Athletic 2024). With the expanded format of 48 teams and 104 matches, the 2026 FIFA World Cup will be the biggest World Cup to date – placing additional physical demand on players and officials, and increasing the risk of extreme heat given the additional matches across a wider range of host locations. Some recent studies have begun assessing heat risk ahead of this tournament. Lindner-Cendrowska et al. (2024) applied an adjusted Universal Thermal Climate Index (UTCI) and concluded that ten out of the 16 host locations are at very high risk of experiencing extreme heat stress. While the UTCI index accounts for thermoregulatory responses in the human body in a way that the WBGT index does not, the latter is the index currently adopted by FIFA in their football heat guidelines and is arguably more practical due to its simplicity, well-defined thresholds, and widespread adoption in occupational and sporting safety protocols and risk assessments. Gouttebarge et al. (2023) and Craig and Karabas (2024) applied the WBGT index to the 2026 FIFA World Cup and noted the risk of extreme heat at several of the host locations. The former presented their findings for a 3-hour afternoon period and 3-hour evening period, and the latter at a reduced daily temporal resolution. Our study aims to investigate the risk of extreme heat to the scheduling of football matches for the 16 host locations at the 2026 FIFA World Cup, using hourly meteorological data to calculate WBGT for the period 2003–2022. Our study therefore represents an advance on the two aforementioned WBGT analyses and demonstrates novelty in the calculation of WBGT at an enhanced temporal resolution, enabling a more thorough examination into the potential impacts of heat stress during different hours of the day that may help inform any potential interventions needed in the scheduling of matches at the 2026 FIFA World Cup tournament.

## Materials and methods

### Study area

**Fig. 1:**
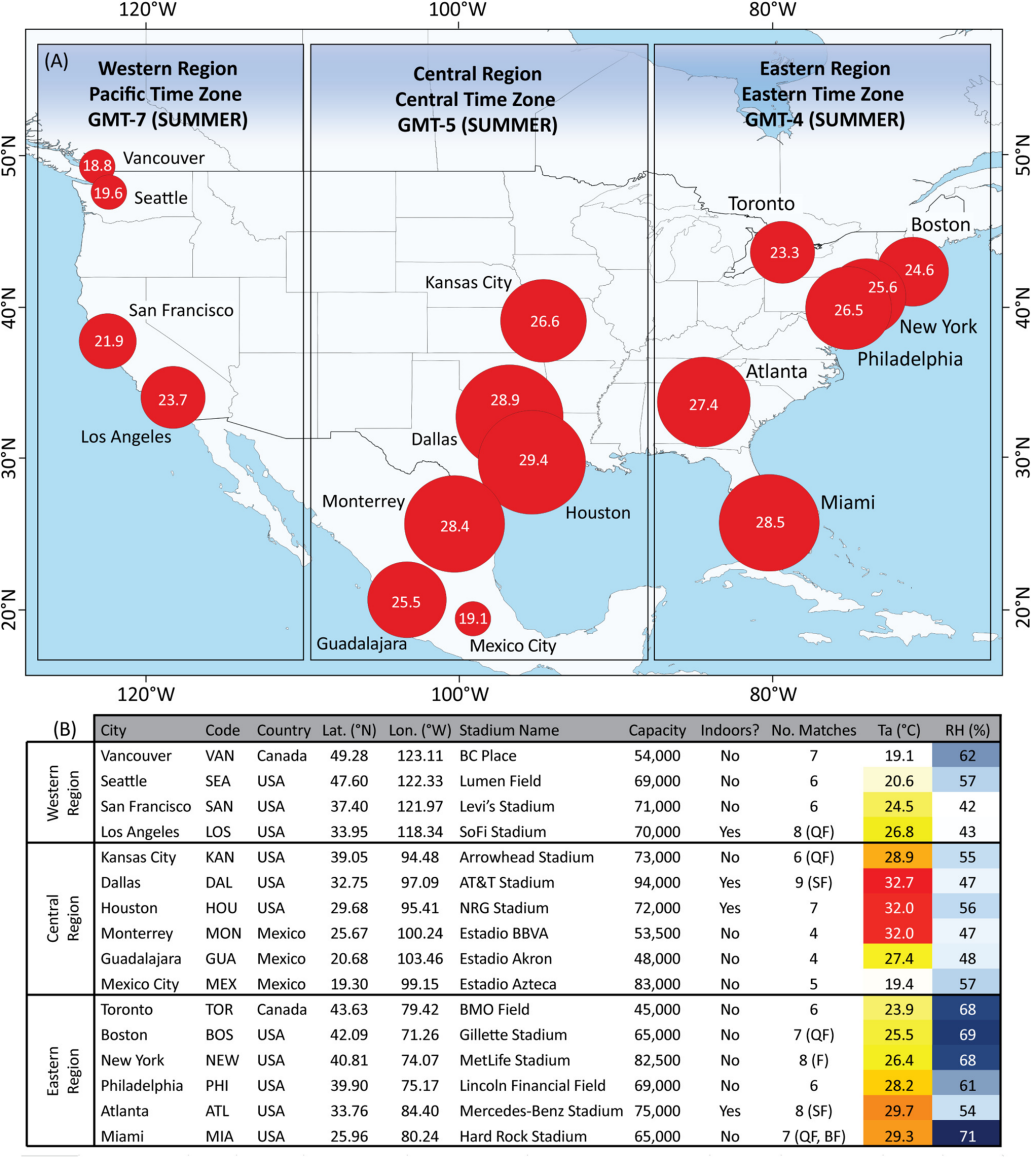
**A **Geography of the 16 host locations, with red bubbles showing mean June and July WBGT during the afternoon hours 12pm-5pm for the 16 host locations during an average year (the mean WBGT across the period 2003–2022). **B **Geographical and sporting details pertaining to the 16 host locations. Ta: Air Temperatures; RH: Relative Humidity – both for an average year with the same averaging hours and years as above. QF: Quarter-Finals; SF: Semi-Finals; BF: Bronze Final; F: Final)

The geographical focus of this study is North America – with 16 stadia across 16 different locations in Canada, USA and Mexico selected to host football matches at the 2026 FIFA World Cup, lying between longitudes 71.3° – 123.1°W and latitudes 19.3° – 49.3°N (Fig. 1). The 16 locations are divided into three geographical (time) zones – the Western Region (Pacific Time Zone, Summer GMT-7), the Central Region (Central Time Zone, Summer GMT-5), and the Eastern Region (Eastern Time Zone, Summer GMT-4). Climate is hugely varied between and within these geographical zones (as shown in Fig. 1), with humid subtropical climates across much of the Central and Eastern Region (with the exception of the subtropical highland climate of Mexico City and the tropical monsoon climate of Miami); and the temperate, oceanic climates of the Western Region – with a clear north-south temperature gradient apparent across all regions. The 16 host locations have stadium capacities ranging from 48,000 at Estadio Akron in Guadalajara to the 94,000 AT&T Stadium in Dallas. A minimum of four matches will be played at each stadium, and a maximum of nine matches at the aforementioned AT&T Stadium. The Final will be held at the MetLife Stadium in New York on 19 July 2026, with crucial knockout matches played during the hottest weeks of July in many of the host locations with highest heat risk – including Kansas City, Dallas, Atlanta and Miami (FIFA 2024). Four of the 16 host locations – Los Angeles, Dallas, Houston and Atlanta – have retractable rooves that form an indoor air-conditioned environment (Stadiums of Pro Football 2024). This considerably alleviates the risk of extreme heat to players, match officials and fans at these venues.

### Datasets

For the 0.5° x 0.5° grid square containing the stadium of each host location, ERA5 reanalysis data were downloaded for temperature, dew point temperature, solar radiation, and wind speed, at an hourly temporal resolution from 2003 to 2022 through the Copernicus Climate Data Store (https://cds.climate.copernicus.eu/). This time period was chosen to represent a very recent climate baseline, providing a set of observations representative of what might be expected at the 2026 World Cup. Downloaded solar radiation data (surface solar radiation downwards) were converted from the downloaded unit (J m^−2^) to w m^−2^ using Eq. 1.

1 $$\:sr=\frac{\text{s}\text{r}\:\left(\text{J}{\:m}^{-2}\right)}{3600}$$

where sr = solar radiation (w m^−2^) and 3600 represents the number of seconds in one hour (Boiley and Wald 2015). Wind speed data were downloaded as the 10 m u-component of wind (zonal) and 10 m v-component of wind (meridional) and converted to total wind speed using Eq. 2 (ECMWF 2024).

2 $$\:ws=sqrt({u}^{2}+{v}^{2})$$

where ws = wind speed (m s^−1^); u = zonal wind (m s^−1^); v = meridional wind (m s^−1^). WBGT is the weighted sum of the natural wet bulb temperature (T_*w*_), the globe temperature (T_*g*_), and the dry bulb (ambient) temperature (T_*a*_) (Budd 2008):

3 $$\:WBGT=0.7{T}_{w}+0.2{T}_{g}+{0.1T}_{a}$$

Separate models are required to calculate T_*w*_ and T_*g*_ to accurately simulate WBGT. We calculated WBGT using the WBGT Liljegren Model (Liljegren et al. 2008) on an hourly basis using the above meteorological variables as inputs, and executed using the HeatStress package in R (https://rdrr.io/github/anacv/HeatStress/). The WBGT Liljegren model meets the best standards for WBGT models (Lemke and Kjellstrom 2012) and is accurate to within 1 °C at a variety of study sites with different climates (Hosokawa et al. 2018). Patel et al. (2013) and Lemke and Kjellstrom (2012) deemed the Liljegren model superior to other WBGT models for outdoor use.

### Methods

WBGT was calculated using the ERA5 reanalysis data described above for a 0.5° x 0.5° grid cell encompassing each host stadium. These values represent outdoor conditions in the surrounding region rather than the precise meteorological conditions within each stadium. We calculated the mean WBGT for the months of June and July (the months the 2026 World Cup will be played) using (1) the entire 20 year record from 2003 to 2022 (herein referred to as ‘an average year’), and (2) the year with maximum June and July WBGT between 2003 and 2022 for each host location (herein referred to as ‘a hot year’). The former reflects average conditions over a very recent climate baseline period while the latter reflects a more extreme year – an important addition given that record breaking temperatures in recent years mean the statistical probabilities of extremes are increasing over time (Fischer et al. 2021). We then calculated the percentage of days across June/July where WBGT exceeded three threshold values: 26 °C, 28 °C and 32 °C. We chose these thresholds on the basis of extreme heat guidelines recommended by Football Australia (Football Australia 2024) and in Gouttebarge et al. (2023). They recommend cooling breaks for players when WBGT is > 26 °C and < 28 °C, and delay or postponement when WBGTs > 28 °C. The more extreme 32 °C threshold was selected given this is the threshold FIFA adopt in their heat guidelines, whereby mandatory cooling breaks are provided in each half as a mitigation strategy, with the added possibility of match delay or postponement at the discretion of match organisers (Kramer and Dvořák, 2015). Exceedances are expressed as percentages – as used in several studies (e.g. Willett and Sherwood 2012), given the ease of interpretation compared to number of days out of 61 during June and July.

## Results

### Spatial variability

Figure 1 shows that WBGTs are generally highest in the Central Region, most notably in the Texas cities Dallas and Houston and the Mexican city of Monterrey – where mean June and July afternoon temperatures (WBGTs) between 12pm and 5pm exceed 32 °C (28 °C). Mean afternoon temperatures (WBGTs) exceed 27 °C (25 °C) in Kansas City and Guadalajara, while Mexico City is the low outlier with temperatures and WBGTs just over 19 °C. In the Eastern Region we see a clear north-south gradient, with warm mean June and July afternoon temperatures ranging between 23.9 °C and 26.4 °C, and high relative humidity > 65% from Toronto southwards to New York – resulting in WBGTs up to 25.6 °C. Philadelphia, Atlanta and Miami are progressively hotter, with temperatures (WBGTs) between 28.2 °C and 29.7 °C (26.5 °C and 28.5 °C) and particularly high humidity of 71% in Miami. Lowest WBGTs are generally found in the Western Region. Vancouver and Seattle have temperate, oceanic climates with modest mean June and July afternoon temperatures (WBGTs) < 21 °C (20 °C) and fairly high relative humidity ~ 60%, contrasting with the warmer and drier climates of the California host locations with mean June and July afternoon temperatures (WBGTs) of 24.5 °C and 26.8 °C (21.9 °C and 23.7 °C) in San Francisco and Los Angeles respectively, and much lower relative humidity < 45% in both locations.

### Diurnal variability

**Fig. 2:**
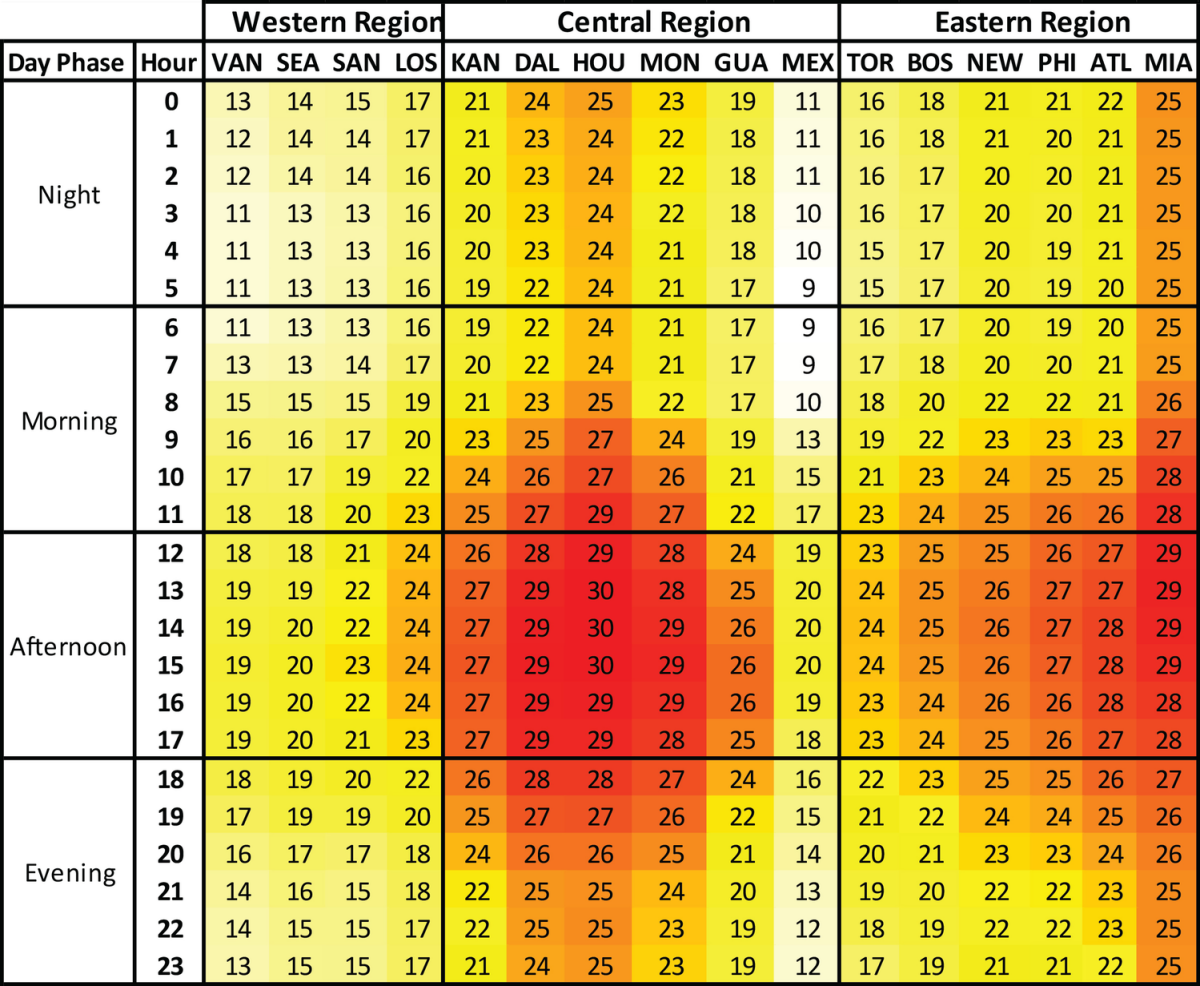
Mean hourly June and July WBGT for the 16 host locations during an average year (the mean WBGT across the period 2003–2022)

When we examine hourly WBGTs in Fig. 2 we see similar diurnal trends across all 16 host locations, with WBGTs steadily rising through the morning, peaking in the afternoon, falling away in the evening and through the night, and reaching their lowest point just after sunrise. In an average year mean June and July WBGTs surpass 26 °C at six locations and 28 °C in Houston and Miami by late morning (11am). In Fig. SI1 we see that in a hot year WBGTs by 11am exceed 26 °C in half the host locations, and above 28 °C in a quarter. By the afternoon WBGTs in an average (hot) year peak at 30 °C (31 °C) in Houston, 29 °C (30 °C) in Miami, Dallas and Monterrey, and 28 °C (29 °C) in Atlanta. In a hot year, afternoon WBGTs also reach 29 °C in Kansas City and Philadelphia, and 28 °C in Boston and New York. By early evening time (6pm), WBGTs in an average year remain above 26 °C at six locations, and above 28 °C in Dallas and Houston. In a hot year the 26 °C threshold is surpassed at eight locations and the 28 °C threshold at six locations by 6pm. Later into the evening (9pm), WBGTs of 26 °C are not surpassed anywhere in an average year, while Dallas, Houston and Miami cross these levels in a hot year.

### Threshold exceedances

**Fig. 3:**
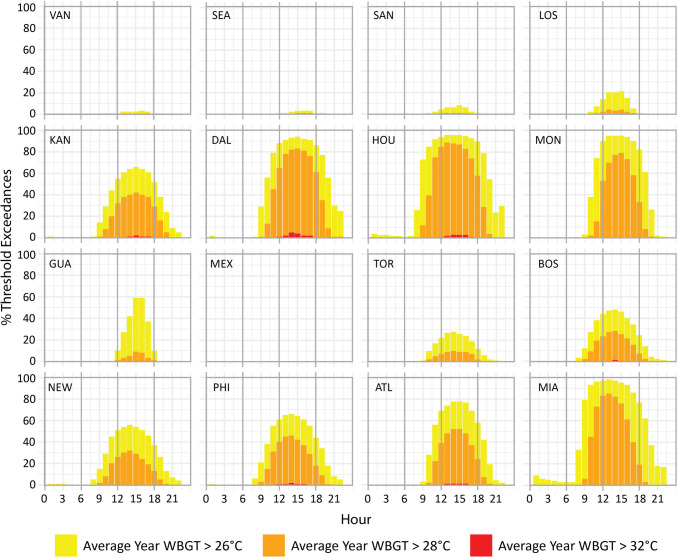
Mean hourly June and July WBGT threshold exceedances (%) at 26 °C (yellow), 28 °C (orange) and 32 °C (red) during an average year (the mean WBGT across the period 2003–2022)

**Fig. 4:**
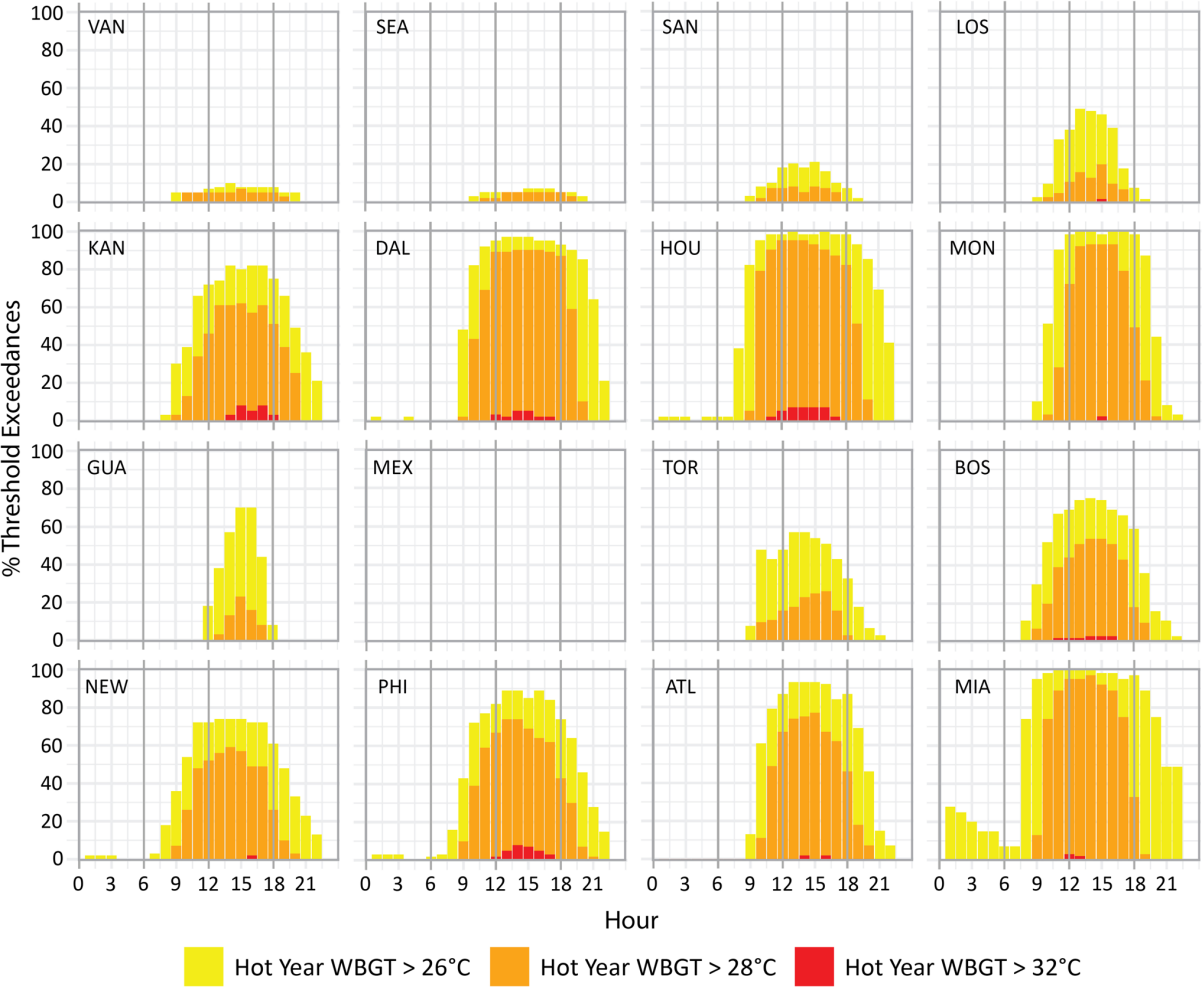
Mean hourly June and July WBGT threshold exceedances (%) at 26 °C (yellow), 28 °C (orange) and 32 °C (red) during a hot year (the year with maximum WBGT across the period 2003–2022)

When we examine the percentage of days in June and July that exceed the selected WBGT thresholds (Figs. 3 and 4) only Mexico City fails to cross the lowest 26 °C threshold in an average year and in a hot year. In an average (hot) year WBGTs above 26 °C are exceeded on > 90% (95%) of June and July days in Dallas, Houston, Monterrey and Miami, > 70% (90%) in Atlanta, > 60% (80%) in Kansas City and Philadelphia, > 50% (70%) in Guadalajara and New York, > 40% (70%) in Boston, > 20% (40%) in Los Angeles and Toronto, and < 10% (25%) in Vancouver, Seattle and San Francisco. While peak exceedances for every location occur in the afternoon, > 50% (80%) of June and July days exceed 26 °C by 10am in Dallas, Houston and Miami in an average (hot) year, remaining above this threshold at the same locations > 50% (75%) of the time by 8pm. Exceedances in an average (hot) year above 25% (35%) in Kansas City, Monterrey, Boston, New York, Philadelphia and Atlanta by 10am are also notable, as are values > 10% (40%) by 8pm in the same locations (apart from Boston).

Mexico City is again the only host location with no days that exceed the 28 °C threshold in an average or a hot year, while Vancouver also fails to cross this threshold in an average year. Exceedances are again greatest in Dallas, Houston and Miami, with > 80% (90%) of June and July days exceeding 28 °C in an average (hot) year. Exceedances > 70% (90%) occur in Monterrey, > 40% (60%) in Kansas City, Philadelphia and Atlanta, > 20% (50%) in Boston and New York, > 10% (25%) in Toronto, and < 10% (25%) in Seattle, San Francisco, Los Angeles, Guadalajara, and (Toronto). Outside afternoons, exceedances above 28 °C by 10am occur > 35% (70%) of June and July mornings in Houston and Miami in an average (hot) year. Exceedances > 10% (35%) in Dallas and Philadelphia are also noteworthy by 10am. Kansas City, Dallas and Houston experience the most exceedances in the evening, with < 10% (> 10%) of June and July WBGTs remaining above the 28 °C threshold by 8pm.

Six host locations (Kansas City, Dallas, Houston, Boston, Philadelphia and Atlanta) experience June and July days that exceed the 32 °C threshold in an average year – all 5% or less of the time, with the highest exceedance of 5% in Dallas at 2pm. An additional four locations exceed the same threshold in a hot year (Los Angeles, Monterrey, New York and Miami). The greatest number of exceedances in a hot year (8%) are found in Kansas City (3pm-5pm) and Philadelphia (2pm), while other locations range between 2 and 7%. There are no exceedances of the 32 °C threshold anywhere outside afternoon hours in an average year, with just 2% exceedances at 11am in Houston and Boston, and 3% at 6pm in Kansas City in a hot year.

## Discussion

This study aims to investigate the risk of extreme heat to the scheduling of football matches for the 16 host locations at the 2026 FIFA World Cup. This section interprets the results and discusses their implications.

### Spatial and diurnal variability in extreme heat risk

Extreme heat risk follows the spatial patterns we would expect based on regional climate classifications as outlined in “Study area” section, with highest WBGT and threshold exceedances in the Central Region and southerly host locations in the Eastern Region. The Central Region is characterised by humid subtropical climates. Extreme heat in this region reflects the northward movement of the subtropical high pressure belt into these latitudes in summer. This promotes anticyclonic weather in coastal areas and allows hot air to be drawn further north into the continental interior. Without any moderating influences of surrounding oceans this allows the Central Region to heat up considerably (NOAA 2024b). The anomaly in the Central Region is Mexico City, with much lower temperatures reflecting its high elevation and subtropical highland climate (Beck et al. 2023). The Eastern Region lies primarily in the humid subtropical zone, where dominant prevailing westerly winds limit maritime influences, resulting in a more continental temperature regime and a distinct north-south gradient (Barry and Chorley 2010). This gives the region warm mean June and July afternoon temperatures, with particularly high humidity in Miami reflecting its tropical monsoon climate (Beck et al. 2023). Generally lowest WBGTs in the Western Region reflect the moderating influence of the Pacific Ocean and cooler summer sea surface temperatures compared to similar latitudes off western Europe, again with a clear north-south gradient (Barry and Chorley 2010). Typical diurnal patterns relating to solar radiation and the principles of heat transfer are observed in all 16 host locations, with highest summer WBGTs in the mid-afternoon and lowest WBGTs in the early morning just after sunrise (Aguado and Burt 2014).

### Could actual heat risk be higher?

Exceedances of WBGT thresholds in this study were first calculated for the mean of June and July from 2003 to 2022. While this recent baseline offers representation for the expected climate at the 2026 FIFA World Cup, it is important to highlight that record breaking temperatures in recent years mean the statistical probabilities of extremes are increasing over time (Fischer et al. 2021). Two of the three hottest June-July periods in the contiguous USA between 2003 and 2022 were experienced in the last two years of our chosen averaging period, with 2021 and 2022 warmer than the 20 year mean by 0.8 °C and 0.6 °C respectively (NOAA 2024a). Hotter conditions in 2021 are in part associated with a heatwave in western North America, among the most extreme events ever recorded globally in terms of how far it sat outside natural variability (Thompson et al. 2022). The results of our study for an average year therefore must be interpreted as the minimum likely scenario, as these WBGTs and their exceedances may actually be conservative estimates of summer temperatures at the 2026 FIFA World Cup. Results for a hot year are presented as a less likely possibility given their more extreme nature and yet it is also possible WBGTs and their exceedances could be higher given that temperature records continue to be broken, often on consecutive years, in a warming world (Fischer et al. 2021).

### How will extreme heat impact players and match officials?

Extreme heat posed health concerns at the 2024 Copa América hosted by USA – a portent for the 2026 FIFA World Cup given the similar timing and host locations. Guatemalan match official Humberto Panjoj had to be withdrawn from a match between Canada and Peru in Kansas City after collapsing on the pitch with ambient air temperatures reaching 33 °C and relative humidity exceeding 50% (Kloke and Burrows 2024). Using the same WBGT model applied in this study, this equates to a WBGT of ~ 27.5 °C. Our results show that 14 out of the 16 host locations experience June and July WBGTs exceeding this value in an average year. Four locations exceed these levels on > 50% of June and July days in an average year, and nine in a hot year. Viewed within the context of the extreme heat issues faced at the 2024 Copa América, these results highlight the potentially serious concern of extreme heat for the health of players and match officials at the 2026 FIFA World Cup. The “Introduction” section outlined how extreme heat impacts the physical and technical performance of players. One particularly relevant study showed that the total number of sprints and high intensity distance covered at the 2014 FIFA World Cup in Brazil was lower in matches played in WBGTs beyond 28 °C than matches below this threshold by ~ 10% and 24.8 ± 2.8 m/min/player respectively (Nassis et al. 2015). These threshold exceedances affected a quarter of matches (16 out of 64) played during the 2014 FIFA World Cup. Our findings reveal up to 88% (92 out of 104 matches) could be played under the same high environmental stress category in an average year, reflecting the larger potential for a drop in player’s physical output, for the same or greater physiological responses, at the 2026 FIFA World Cup. Conversely, Nassis et al. (2015) found that the rate of successful passes was 3.2% higher at WBGTs above 28 °C, indicating the possibility that technical performance could increase at higher levels of extreme heat.

### Practical implications

Our analysis shows that afternoon kick-off times present the greatest risk of extreme heat to the scheduling of football matches at the 2026 FIFA World Cup. In Dallas, Houston, Monterrey and Miami we can expect at least three quarters of June and July afternoons to exceed the 28 °C WBGT threshold in an average year. In a hot year we could expect at least half of June and July afternoons to cross the same threshold in the additional host locations of Kansas City, Boston, New York, Philadelphia and Atlanta – highlighting that extreme heat risk in the afternoons is a serious concern for more than half the host locations. Three of these nine host locations are equipped with an indoor, air-conditioned environment, so the six locations of greatest concern for heat risk become Monterrey, Miami, Kansas City, Boston, New York and Philadelphia. These findings are similar to Hosokawa et al. (2018), who showed that WBGTs during the 2020 Summer Olympic football tournament in Japan could exceed 30 °C in 40–50% of late mornings and early afternoons, while early morning and late afternoon games substantially reduced the likelihood of experiencing WBGTs > 30 °C. The reality of this assessment could be seen when the Women’s Olympic Football Final between Canada and Sweden was rescheduled from an 11am kick-off time to the later time of 9pm due to concerns over heat and humidity (BBC 2021). Given the afternoon heat risk at many of the host locations in this study, there is a climatically sound argument for scheduling kick-off times outside the peak afternoon heat in the host locations with highest heat risk – notably the six locations identified above. The effect of this can clearly be seen for Miami – where WBGTs of 28 °C in an average year are exceeded only 7% of the time when averaged between the hours of 6pm-9pm compared to 80% between 12pm-3pm.

### Limitations and future work

The ERA5 reanalysis data – used in this study to estimate WBGT – has been known to underestimate extreme heat (e.g. Raymond et al. 2020), meaning our results may be conservative estimates of actual conditions. Differences between ERA5 data and meteorological conditions within stadia include overestimated solar radiation due to shading from seats and stands, and overestimated wind speeds due to enclosure by the stadium and because ERA5 wind speed data are provided at a 10 m elevation rather than at pitch level (Hersbach et al. 2020). Use of local weather stations may provide improved estimates of heat risk (e.g. Mistry et al. 2022), but the stations themselves may be located in microclimates unrepresentative of conditions within the stadium (e.g. Davey and Pielke Sr 2005). This underlines the urgent need to instrument modern stadia to enable more detailed estimates – and forecasts – of heat risk within venues. This study uses WBGT thresholds from football governing bodies that relate to potential interventions to the scheduling of matches (e.g. cooling breaks or postponement), but it is unclear whether these thresholds are based on any analysis of physiological impacts on players and match officials. Future studies could use physiological models such as Predicted Heat Strain (PHS) for a more detailed evaluation around the potential motor-cognitive impact of heat stress on players and match officials (e.g. Piil et al. 2021). The physical exertion involved in sport means athletes may be at higher risk of EHI than spectators (Brocherie et al. 2015; Olya 2019), yet the impact for the latter group cannot be underplayed considering the tens of thousands of spectators who attend major sporting events – often coming from locations where they are unacclimatised (Vanos et al. 2019). Spectators at major sporting events are likely standing in crowded areas for at least 2–3 h (often in direct sunlight), with overcrowding further increasing the heat load from the human metabolic output (Stewart and Kennedy 2016) and decreasing airflow (Howarth et al. 2011). Future studies could investigate the most appropriate WBGT thresholds representative of football fans and compute these to consider the extent of heat risk for the many thousands of people this category represents. Future work could also apply climate model simulations for 2026 to consider a wider range of possibilities beyond the average and hot year computations made in this study. Furthermore, climate scenarios could be analysed for the 16 host locations further into the future to ascertain longer-term heat risk that may assist with future planning, e.g. Smith et al. (2016) with respect to the future of the Summer Olympics – including the possibility of reorganisation of future football tournaments in North America to more suitable locations or times of the year.

## Conclusions

This study has shown that extreme heat risk may necessitate potential interventions to the scheduling of football matches at the 2026 FIFA World Cup. Almost 90% of host locations experience WBGTs in an average year exceeding thresholds beyond which some football governing bodies would recommend cancellation or postponement, with over 50% of host locations exceeding these levels more than half the time in a hot year. Most of this heat risk falls in the afternoon hours, with substantially lower risk in the evenings. Practical implications of these results include the climatic argument to schedule kick-off times outside peak afternoon heat for the host locations with highest heat risk for which no indoor air conditioning exists – particularly Monterrey and Miami, but also potentially Kansas City, Boston, New York and Philadelphia. Our study highlights the importance of carefully assessing heat risk ahead of major sporting events to help inform any potential interventions needed in the scheduling of matches and competitions in a warming climate.

## Supplementary information

Below is the link to the electronic supplementary material.

ESM 1 Mean hourly June and July WBGT for the 16 host locations during a hot year (the year with maximum WBGT across the period 2003–2022). (JPG 1.86 MB)

## Data Availability

All data used in this study are freely available to download via the links provided in the “Materials and methods” section.

## Declarations

All authors have read, understood, and properly complied with the statement in the Instructions for Authors and are aware that, with minor exceptions, no changes in authorship can be made after submission.
