# Supplementary figures and images for: Extreme heat risk and the potential implications for the scheduling of football matches at the 2026 FIFA World Cup
Source: Int J Biometeorol. 2025 Jan 25;69(4):753–63. doi: 10.1007/s00484-025-02852-4 (PMC11947059; doi:10.1007/s00484-025-02852-4)

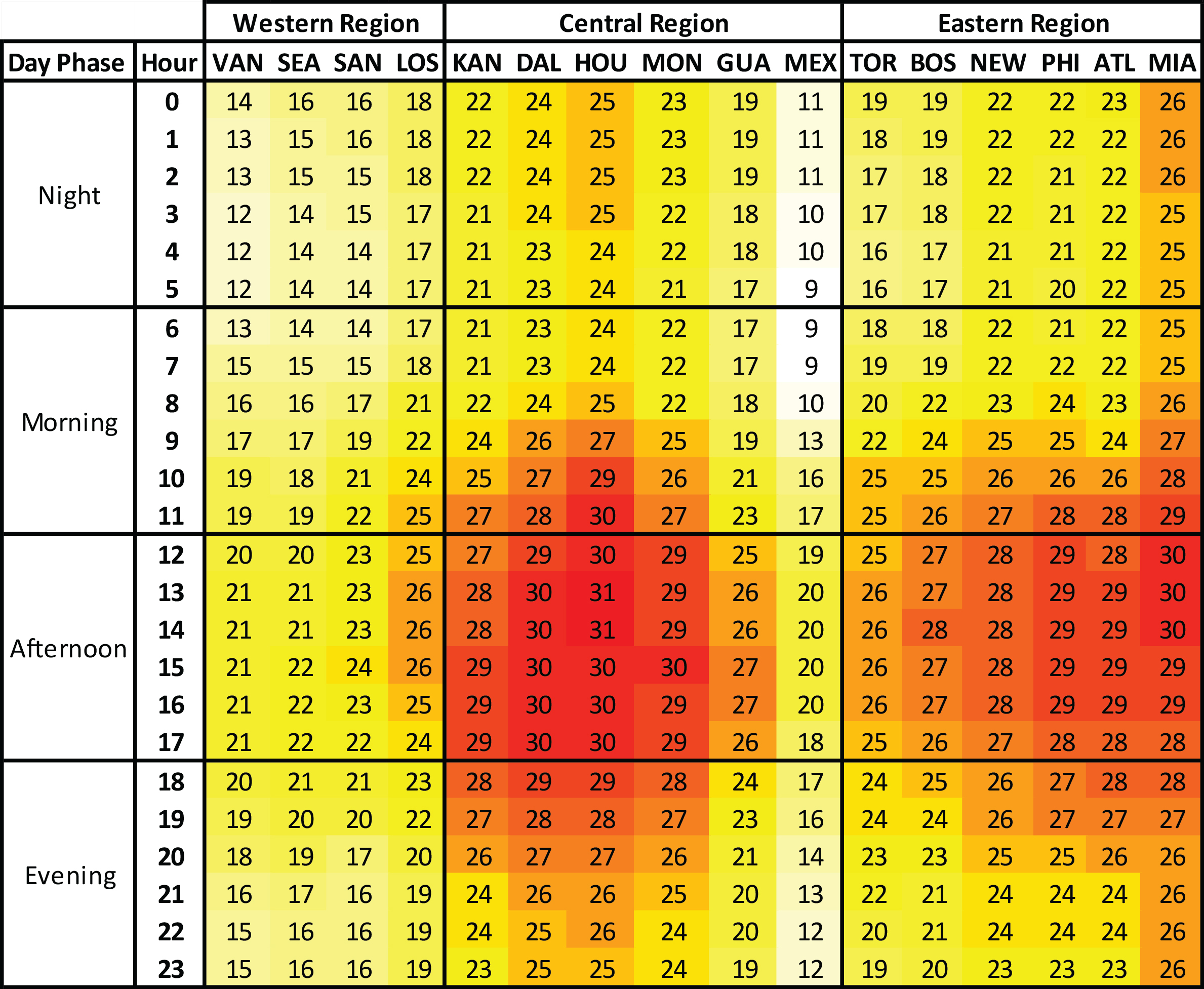

Supplement: Supplementary file 1 — Mean hourly June and July WBGT for the 16 host locations during a hot year (the year with maximum WBGT across the period 2003–2022). (JPG 1.86 MB) [file 484_2025_2852_MOESM1_ESM.jpg]
